# Pain during the first year after scoliosis surgery in adolescents, an exploratory, prospective cohort study

**DOI:** 10.3389/fped.2024.1293588

**Published:** 2024-01-19

**Authors:** Thomas G. de Leeuw, Anneke A. Boerlage, Hanneke M. van West, Jeroen J. M. Renkens, Joost van Rosmalen, Lonneke M. E. Staals, Frank Weber, Dick Tibboel, Saskia N. de Wildt

**Affiliations:** ^1^ Department of Anesthesiology, Erasmus MC-Sophia Children’s Hospital, Rotterdam, Netherlands; ^2^ Department of Neonatal and Pediatric Intensive Care, Erasmus MC-Sophia Children’s Hospital, Rotterdam, Netherlands; ^3^ Department of Orthopedic Surgery, Erasmus MC-Sophia Children’s Hospital, Rotterdam, Netherlands; ^4^ Department of Biostatistics, Erasmus MC, Rotterdam, Netherlands; ^5^ Department of Epidemiology, Erasmus MC, Rotterdam, Netherlands; ^6^ Department of Pharmacology and Toxicology, Radboud Institute for Health Sciences, Radboud University Medical Center, Nijmegen, Netherlands

**Keywords:** scoliosis, chronic postsurgical pain, adolescents, preoperative pain, T-QST

## Abstract

**Objective:**

Approximately 50% of adolescents who have undergone scoliosis surgery still experience severe pain one year postoperatively. We explored the postoperative pain trajectory and the potential value of preoperative Thermal Quantitative Sensory Testing (T-QST) as predictor of chronic postsurgical pain after scoliosis surgery.

**Design:**

Single-center prospective cohort study in adolescents undergoing scoliosis surgery.

**Outcomes:**

Prevalence of chronic postsurgical pain (CPSP) one year after scoliosis surgery and postsurgical pain course during this year. The need for rescue medication and the relationship between pre-operative T-QST, acute pain and CPSP.

**Results:**

Thirty-nine patients (mean age 13.9 years; SD 1.9 years) completed the study. One year postoperatively, ten patients (26%) self-reported pain [numeric rating scale (NRS) score ≥ 4]) when moving and two (5%) when in rest. Four of these patients (10.3%) experienced neuropathic pain. The pre-operative cold pain threshold was lower (*p* = 0.002) in patients with CPSP at 12 months. Preoperative cold and heat pain thresholds were correlated with the number of moderate or severe pain reports (NRS ≥ 4) in the first week postoperatively (*r* -.426; *p* = 0.009 and *r*.392; *p* = 0.016, respectively).

**Conclusions:**

One year after scoliosis surgery, a significant part of patients (26%) still reported pain, some with neuropathic characteristics. Better diagnosis and treatment is needed; our study suggests that T-QST could be further explored to better understand and treat children with this negative outcome.

## Introduction

Chronic postsurgical pain (CPSP) in children and adolescents, defined as surgery-related pain lasting for more than 3 months postoperatively, is increasingly recognized, as witnessed by its inclusion in the International Classification of Diseases, 11th revision (ICD-11) (1, 2). The estimated overall prevalence one year after surgery is approximately 20% (3) and for scoliosis surgery 50% (4).

Potential risk factors for CPSP are the intensity of acute postoperative pain, presence of preoperative pain, and psychological distress such as anxiety, depression or catastrophizing (5–7). In children, parental catastrophizing also predicts CPSP 12 months after surgery (8). Additionally, major orthopedic surgery in general appears to be a main cause for CPSP (9). More specifically studies concerning scoliosis surgery report a prevalence of severe CPSP [numeric rating scale (NRS) score > 7] from 10% to 16% up to 5 years after surgery (10, 11). Spinal fusion surgery often requires prolonged pain treatment directly after postoperative discharge (12). Several risk factors were identified, based on retrospective correlations or single time point correlations. A study in 106 scoliosis patients found that total opioid consumption during the acute postoperative period was not related to pain, pain medication or functional activity 6 months after surgery (13). Preoperative pain, described in 35%–78% of patients, as well as the patient's perception of the severity of the deformation have been found important predictors for CPSP, while there is no clear evidence of a relationship between the magnitude of curve correction and risk of CPSP (10, 14, 15).

CPSP often shares characteristics of chronic neuropathic or nociplastic pain (1), for which continuous preoperative pain over 3 months as well as post-operative morphine consumption have been suggested as predictors (4).

Impaired pain modulation (16) and temporal summation (17) have been suggested as important contributing factors to CPSP. To diagnose impaired pain modulation, and more specifically the related descending inhibition or delayed recovery from central sensitization, Quantitative Sensory Testing could be a promising diagnostic tool (18). It consists of a panel of diagnostic tests, of which Thermal Quantitative Testing (T-QST) is one, used to assess somatosensory function and to define specific sensory profiles. While T-QST is mainly used in research settings to evaluate neuropathic pain, in the clinical setting it may be useful to predict the tendency of patients to develop chronic pain (19, 20). We hypothesized that an increased sensitivity to pain induced by warmth and/or cold expressed as lower pain thresholds for warmth or higher pain thresholds for cold are significantly related to patients' higher risk of acute pain and CPSP after scoliosis surgery.

In this exploratory study, we assessed the postoperative pain trajectory, and the prevalence of CPSP at 1 year after scoliosis surgery. We also explored the predictive value of preoperative T-QST for the development of acute and chronic pain after scoliosis surgery in adolescents.

## Methods

### Study design

This was a single-center, prospective cohort study performed from October 2016 through January 2019 in the Erasmus MC Sophia Children's Hospital, a tertiary children's hospital in Rotterdam, the Netherlands. Since this was not a Randomized Controlled Trial the study was not notified in a trial register, therefore approval for this study was obtained from the hospital's Ethics Review Board (MEC 2015-704).

The manuscript was prepared according the STROBE guidelines (21).

### Subjects

Inclusion criteria were: age between 11 and 18 years old, planned for scoliosis surgery, and signed informed consent by subjects and/or parents/caregivers (according to the Dutch Medical Research Involving Human Subjects Act). Because scoliosis is regularly seen in adolescents with cognitive impairment; we conducted this study in adolescents with and without cognitive impairment if T-QST measurement was possible.

Exclusion criteria were: contra-indication for epidural analgesia, scoliosis surgery combined with other orthopedic surgical procedures. In addition, subjects who would not receive the standardized anesthesia or the protocolled postoperative analgesia were excluded.

### Outcomes

Primary outcome: prevalence of CPSP (defined as surgery-related pain lasting for more than three months postoperatively) at one year postoperatively, defined as a self-reported Numeric Rating Scale (NRS) ≥ 4 in rest or when moving. Furthermore, we followed the course of postoperative pain during the first year after scoliosis surgery.

Secondary outcomes: prevalence of acute postoperative pain, evaluated by pain score (NRS ≥ 4) and need for rescue medication, the relationships between pre-operative T-QST results and chronic postoperative pain at one year, as well as acute postoperative pain.

Furthermore: the magnitude of curve correction and postoperative and chronic pain, the number of times NRS ≥ 4, and the cumulative number and dose of rescue analgesics.

The latter was additionally assessed in patients with cognitively impairment whether this condition influenced postoperative analgesic treatment.

### Measurements

#### Pain scores

For pain assessment, we used the NRS-11, a self-report scale of pain intensity with a rating of 0–10, which has been validated for use in children and adolescents (22–24). NRS scores of 4 and higher indicate substantial pain that should be treated. For cognitively impaired patients with a developmental age under 7 years self reports by means of the Faces Pain Scales-Revised (FPS-R) (25) was used and subsequently converted to a score between 0 and 10 as previously described by Hicks et al. (26).

The “*Douleur Neuropatique en 4 questions*” (DN 4) has been developed in France to assess the presence of neuropathic pain in four questions, and is widely appreciated as most suitable for clinical use (27, 28). Although this scale has been validated for Dutch adults and not for children or patients with a mild cognitive impairment, we used this in the absence of a validated neuropathic scale for pediatric patients (29, 30). For this study we used the DN4 with 7 items (DN4-7) with a cut-off score of 4, consisting of the patients description, character and symptoms of the pain, indicating the presence of neuropathic pain (30). During the follow up visits the DN4 was only used in case of substantial pain (NRS > 4) and a suggested neuropathic character (if the child used descriptors such as “burning”, “electrical sensations”, or “pinpricks”).

To avoid parental influence during measurements parents were asked not to intervene during T-QST measurements and pain assessment.

#### T-QST measurement

To evaluate pain sensitivity, we determined thermal detection- and pain thresholds using the Thermal Sensory Analyzer (TSA type II, Medoc Ltd. Advanced Medical Systems, Ramat, Israel) with a Peltier-based contact thermode. Thermal Quantitative Sensory Testing (T-QST) consists of the Method of Limits (MLI); by which we measured detection thresholds and pain thresholds (reaction time dependent) and the Method of Levels (MLE); measures only detection thresholds (reaction time independent) (31).

For all measurements, the thermode was attached to the thenar of the non-dominant hand. With the MLI, subjects receive four successive ramps of gradually increasing or decreasing temperature and are asked to press a button as soon as a first sensation of pain is perceived.

Pressing the switch results in an automatic recording of the temperature and an automatic reset of the probe temperature to baseline value. With the MLE, subjects receive a series of gradually ascending or descending temperature stimuli with a preset destination temperature, after which the temperature of the probe returns to baseline. Immediately following each stimulus the subject is asked whether he/she feels pain or not.

The baseline temperature was set at 32°C, the minimum temperature at 0°C and the maximum temperature at 50°C. Generally for children with a prolonged reaction time, e.g., due to cognitive impairment or (neuro) muscular disease, the method of levels (MLE) is used, which is reaction-time independent (32, 33). For this study we tested in advance in patients with cognitively impairment or muscular disease whether they were capable either to press the button of the TSA or to react verbally in which case the tester immediately pressed the button. Children who were unable to press the button or react verbally were excluded from the T-QST measurement. To avoid large variation in measurements, the T-QST tests were performed by one researcher (AB) or one pain consultant.

#### Other scores

To assess pain in adolescent idiopathic scoliosis and the impact of surgery, we used the Scoliosis Research Society instrument (SRS-30), the most recent version of a patient-reported outcome questionnaire for this specific population (34–36).

To assess cognitive level, adaptive behavior was established with the Vineland Adaptive Behavior Scales-II (VABS-II). The expanded standardized questionnaire consists of 597 items divided over the five main domains: adaptive behavior composite, communication, daily living skills, socialization and motor skills (37). The parent or caregiver is asked whether the child actually does the particular activity, scored as 0 (never), 1 (sometimes or partially), or 2 (usually). The VABS standard adaptive score is standardized and normed for age and can be used from birth to 90 years of age. The motor skills questions pertain to children aged 6 years or younger, and therefore we excluded these 106 questions. For this study, the Dutch version of the Vineland-II was used, during a telephone interview in the week after informed consent (38). Adolescents who scored minus 2 SD were considered mildly cognitively impaired. Information regarding VABS standardization, validity and reliability can be found in the Survey Forms Manual (37).

### Study procedures

#### Preoperative

Informed consent from parents/legal guardians and/or subjects was obtained preoperatively. To assess a subject's cognitive level, the parents were interviewed by telephone to establish the level of adaptive behavior with the VABS-II. At a preoperative visit, the child's pain history was taken. The DN 4–7 items was part of the standard preoperative pain history. In addition, T-QST was performed as described earlier.

#### Postoperative

During the first seven postsurgical days pain assessment was performed by means of the NRS at least 3 times a day and all analgesics were registered. The participants were called 7 and 14 days after discharge to inform about the use of rescue medication.

**Figure 1:**
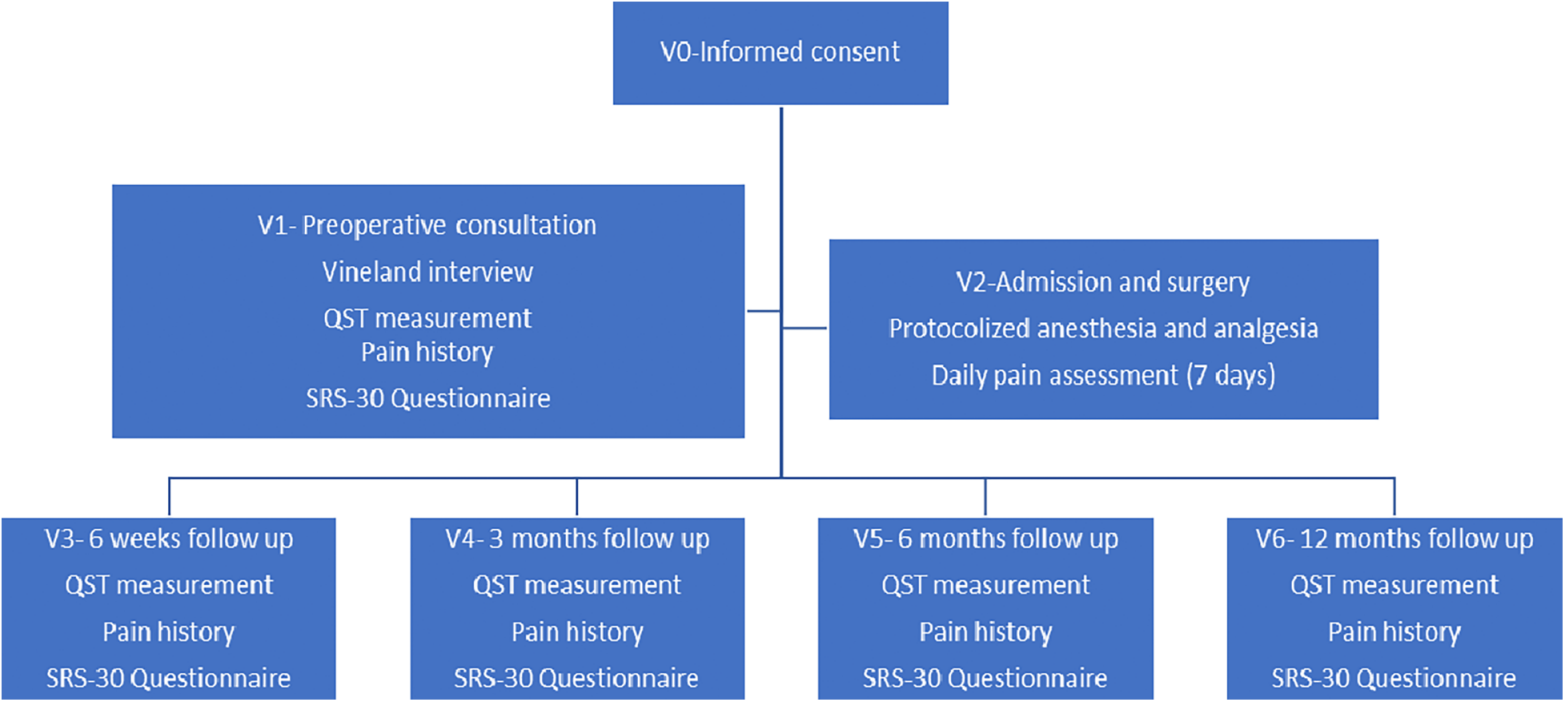
Timeline measurements. V, visit.

Follow up visits took place at 6 weeks, 3 months, 6 months and 12 months, combined with the regular visits to the orthopedic outpatient clinic. During these visits, pain scores were assigned, and T-QST measurement was performed. A measurements timeline is shown in Figure 1.

Before and after surgery, the severity of the deformation (Cobbs angle) was measured independently by two orthopedic surgeons. If opinions differed, consensus was reached after discussion in all cases.

### Standardized anesthesia/pain protocol

Standardized anesthesia and postoperative pain treatment protocols were applied. Induction was performed using 3–4 mg/kg propofol IV or by mask with sevoflurane in O_2_-air mixture after which IV access was introduced. Patients received IV sufentanil 0.3 mcg/kg and rocuronium 0.6 mg/kg followed by tracheal intubation and ventilation. Anesthesia was maintained using IV propofol 6–10 mg/kg/h in combination with IV sufentanil 0.2–0.4 mcg/kg/h and remifentanil 0.1–0.5 mcg/kg/min. When vital signs indicated pain according to the anesthesiologist 1–2 mcg/kg clonidine IV was given.

Monitoring was done by means of ECG, O_2_-saturation, arterial blood pressure, end-tidal CO_2_, central venous pressure, EEG depth of anesthesia monitoring and somatosensory evoked potentials.

Before end of surgery, the orthopedic surgeon placed two epidural catheters under direct vision, one at the cranial side and one at the caudal side of the wound. A loading dose of 0.1 ml/kg ropivacaine 2 mg/ml was given on each catheter followed by a continuous infusion of 0.1 ml/kg of a standard mixture of ropivacaine 2 mg/ml with sufentanil 0.5 mcg/ml. In addition, oral paracetamol 90 mg/kg/day was given in a 6 h interval (tapered off to 60/mg/kg/day on postoperative day four), together with oral diclofenac 3 mg/kg/day in an 8 h interval. Postoperative rescue medication consisted of a bolus of 0.1 ml/kg of the ropivacaine/sufentanil mixture on each epidural catheter, if needed 1 mcg/kg clonidine on each catheter.

In case of technical problems with the epidural catheters an IV PCA-pump of morphine 15 mcg/kg/h with a bolus of 15 mcg/kg and lock-out time of 10 min was started with esketamine 100 mcg/kg/h IV or clonidine 0.1 mcg/kg/h as optional additives. In case of severe muscle pain, oral diazepam 5 mg 3 times daily was started.

At postoperative day four, twice-daily 10 mg oxycodone oral slow release was started followed by discontinuation of the epidural or IV-medication. This regimen was continued for two weeks postoperatively together with paracetamol/diclofenac. Oral oxycodone instant release 5 mg maximum 4 times/day was prescribed as rescue therapy. Patients who were unable to take tablets received a Buprenorphin patch of 5 mcg/h with oral morphine formula 0.3 mg/kg up to 6 times daily as rescue medication for two weeks.

### Statistical analysis

Primary outcomes: descriptive statistics were applied to present the prevalence of CPSP one year after surgery and the course of pain during the first year postoperatively. These data are presented by means of a heat map plot.

Secondary outcomes: Relationships were tested using Spearman's rho, groups were compared using a Mann–Whitney *U* test. Associations between chronic preoperative pain (more than 3 months pain yes/no) and CPSP and the number of times NRS ≥ 4 during the first seven days postoperatively and CPSP were tested with chi-square tests. Univariable ordinal logistic regression by means of the PLUM procedure in SPSS was used to establish whether preoperative T-QST measurement predicts postoperative analgesic requirement. For this analysis, the number of times an analgesic rescue dose was administered during the first week postoperatively was recoded into three categories: 0, no rescue medication; 1, 1 through 4 times; and 2, at least 5 times.

Normally distributed data are presented as mean and standard deviation (SD); non-normally distributed data as median and interquartile range (IQR). Data are summarized using standard descriptive statistics.

All analyses were conducted with IBM SPSS statistics version 25. (IBM-Corp., Armonk, NY, USA) and GraphPad Prism 9.5.1 for MacOS (GraphPad Software, San Diego, CA, USA). All statistical tests were two-sided with a significance level of 0.05.

## Results

### Participants

**Figure 2:**
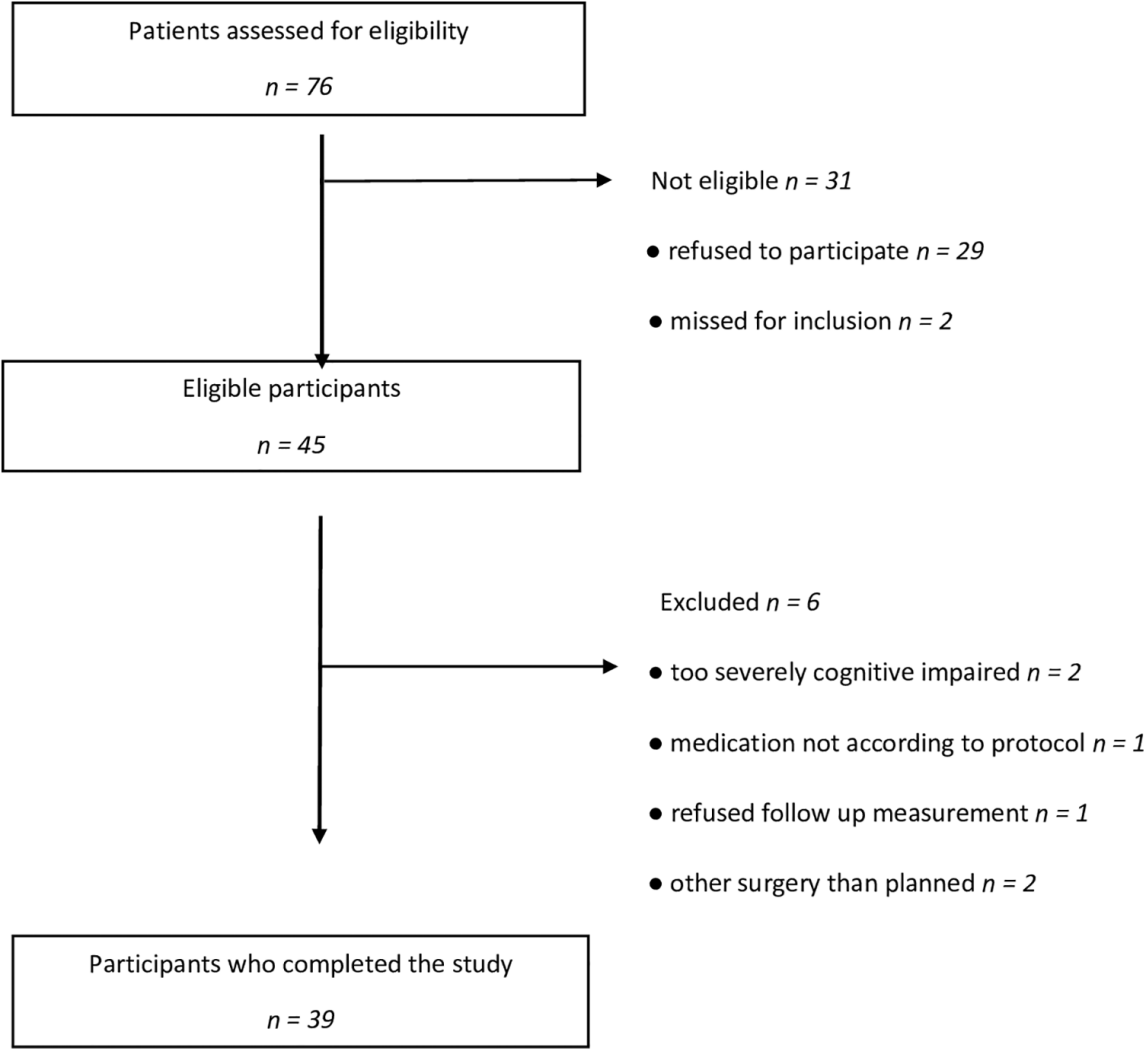
Inclusion of patients.

During the inclusion period, seventy-six patients were eligible for inclusion. Two of them had undergone surgery before they could be included, and 29 others did not give informed consent; thus, 45 participants were enrolled. Six participants did not complete the study for various reasons (See Figure 2).

### Descriptive data

In total 39 patients (26 girls) with a mean age of 13.9 (SD 1.9) years completed the study. Twenty-eight (72%) had been diagnosed with an idiopathic scoliosis; the 11 others with a congenital or musculoskeletal disorder. Seven could be classified as (mildly) cognitively impaired. The included participants with a mild cognitive disability were all able to provide the information and answer the questions, which was confirmed by the caregivers.

In three cases, one postoperative T-QST measurement was missing; in two because the patient missed the appointment, and in one because the patient refused the measurement during follow up at 6 months. For one cognitively impaired patient T-QST measurements were not possible and in 2 of these patients it was impossible to assess a DN4-7 items questionnaire.

Preoperatively, 35 (89.7%) participants reported pain of which 7 reported an NRS of 4 or higher with a median of 6 (IQR 4–7). In all cases participants suffered from dorsal pain located in the region of the scoliosis without a particular irradiation pattern. Their median NRS during rest was 2 (IQR 0–5) and during activities 8 (IQR 5–8). (See Table 1 for the Baseline characteristics).

**Table 1:** Baseline characteristics.

	*N* (%)
Patients	39 (100)
Female	26 (67)
Age	Mean 13.9 (1.9 SD)
Idiopathic scoliosis	28 (72)
Vineland; ABC SD < −2	7 (18)
	Median (IQR)^a^
Cobb's angle	66 (58–77)
Pain intensity during the interview	0 (0–8)
Pain during rest (last week)	0 (0–5)
Pain during movement (last week)	5 (0–9)
Pain duration	N (%)
No pain	5 (12.8)
Less than 3 months	6 (15.4)
3–12 months	8 (20.5)
Longer than 12 months	20 (51.3)
Frequency of pain	N (%)
Less than once per month	3 (7.7)
Once per month	1 (2.6)
2–3 times per month	3 (7.7)
Once a week	6 (15.4)
Twice a week	12 (30.8)
Daily	14 (35.9)
Sleep disturbance	12 (30.8)
Wakeup due to pain	7 (17.9)
DN4score ≥ 4	10 (25.6)
Educational level	
Special educational level	11 (28.2)
Elementary school	3 (7.7)
Average educational level	17 (43.6)
Higher educational level	8 (20.6)
Comorbidities conform the ICD-10^b^	*N* = 18
Neoplasms	1 (3.8)
Endocrine, nutritional and metabolic diseases	2 (7.7)
Mental and behavioral disorders	9 (34.6)
Diseases of the nervous system	1 (3.8)
Diseases of the eye and adnexa	1 (3.8)
Diseases of the respiratory system	1 (3.8)
Diseases of the digestive system	1 (3.8)
Diseases of the musculoskeletal system and connective tissue	6 (33.3)
Congenital malformations, deformations and chromosomal abnormalities	4 (15.4)

^a^ IQR = interquartile range.

^b^ More than one comorbidity per child possible.

An overview of median pain scores and T-QST per postoperative visit at our outpatient pain clinic can be found in Supplementary S1 and S2.

### Primary outcomes

**Figure 3:**
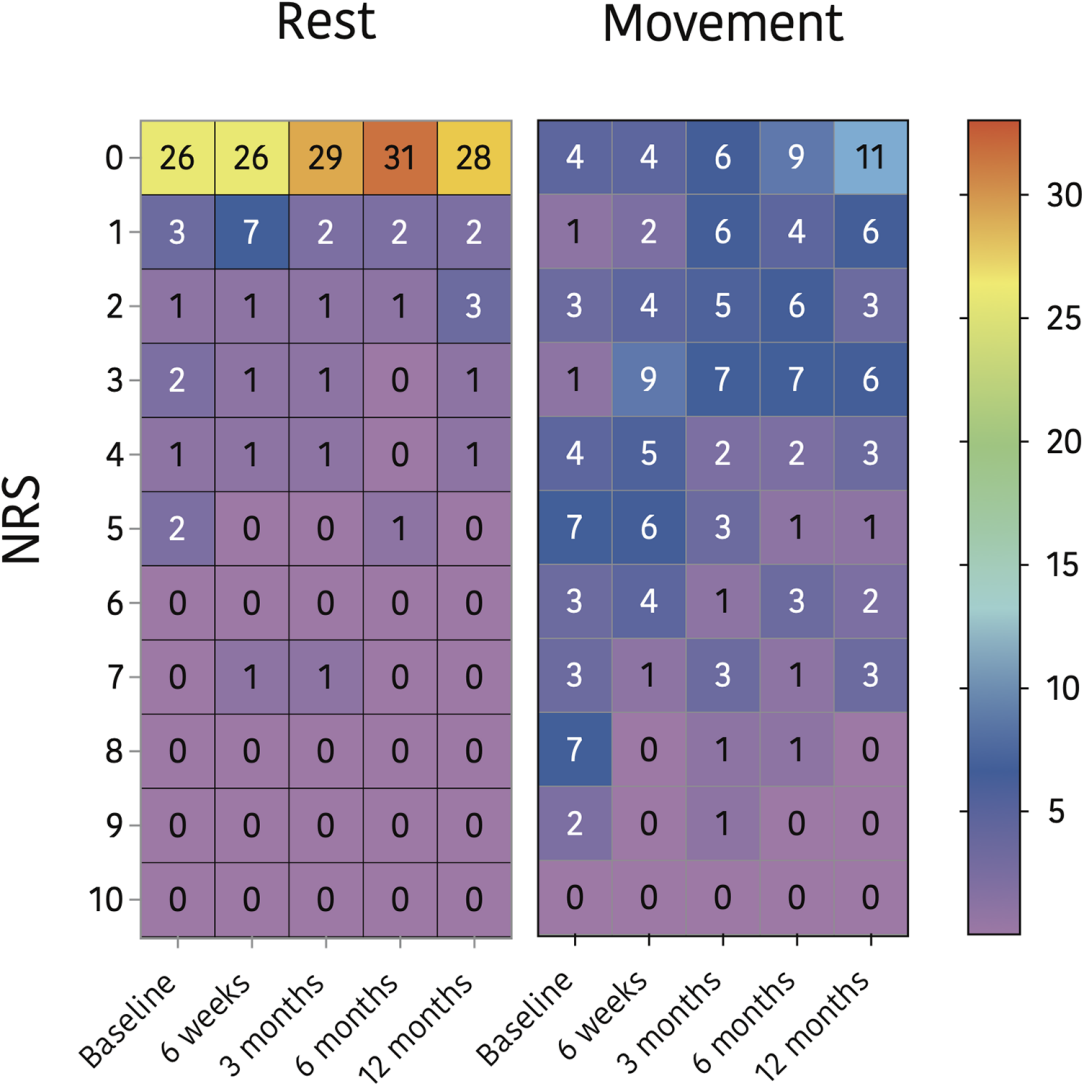
Heat map plot of the postoperative pain course.

One year postoperatively, 10 participants (26%) still suffered from CPSP, in all 10 during movement, and in two of them (5%) at rest, median NRS 6 (IQR 4–7) during movement. Of the 10 children with a DN4-7 items score of ≥4 prior to surgery 2 still scored ≥4 after 12 months. Two other patients developed postsurgical neuropathic pain resulting in a 10.3% prevalence of neuropathic pain one year postoperatively. (See Figure 3).

### Secondary outcomes

#### Postoperative pain and analgesics

During the first postoperative week (days 0–7), a total of 1,118 NRS scores were recorded [median per patient 30 (IQR 23–33)]. Thirty-five participants reported in total 400 NRS scores ≥ 4 [median 9 (IQR 3–15)] and 78 NRS scores ≥ 7 [median 2 (IQR 0–4)]. Eighteen participants, who reported at least once an NRS ≥ 4, received rescue medication. Of the seven cognitively impaired adolescents, one reported an NRS ≥ 4 once and received rescue medication, while three others received rescue analgesics not based on an NRS ≥ 4.

During the first three postoperative days, twelve participants (31%) reported pain, despite the administration of epidural analgesia, and received a clonidine epidural bolus (1 mcg/kg) as rescue medication; four others needed conversion to PCA morphine. Four (10%) other children received IV clonidine continuously in addition to epidural analgesia, and one received esketamine IV continuously in addition to epidural analgesia. Thirteen (33%) participants developed myalgia postoperatively, which was treated with diazepam. After removal of the epidural catheters and during admission, twenty-seven participants received on average 4 doses (IQR 3–7) oral oxycodone immediate release (5 mg) as rescue medication besides the standard oral oxycodone slow-release according to protocol.

#### Pain after discharge

During fourteen days after discharge, nineteen participants used oxycodone immediate release median 6 times a day (IQR 2–15) besides the standard oxycodone slow release, up to a maximum 4 weeks post-discharge.

Based on the SRS-30 outcome at six weeks follow up, twenty-four participants (69%) had experienced pain during the past month, and five of them used mild analgesics on a regular basis. An indication for persistent pain was not found in the group of children with cognitive impairment.

#### Relationship of clinical characteristics and CPSP at one year

All 10 participants diagnosed one year postoperatively with CPSP had reported a preoperative NRS of ≥4, compared to 4 out of the 29 participants without CPSP.

All ten had reported an NRS ≥ 4 at least once during the first postoperative week, and eight of them even an NRS ≥ 7.

#### T-QST measurements and chronic pain

The preoperative cold pain threshold in participants with CPSP one year postoperatively, was significantly lower (less sensitive) than that in children without CPSP (NRS ≥ 4; median 8.0 (IQR 0.8–16.0) vs. NRS < 4; median 19.0 (IQR 12.7–22.5), *p* = 0.002). The other T-QST measurements did not differ between those with and those without CPSP (Table 2).

**Table 2:** Thermal-QST measurement and pain at 12 months.

Method of limits	*N*	NRS < 4 Median (IQR) *N* = 27/28	*N*	NRS ≥ 4 Median (IQR) *N* = 10	*P*-value^a^
Cold sensitivity (^o^C)	27	30.2 (29.2–30.6)	10	30.5 (30.1–31.3)	0.044
Warmth sensitivity (^o^C)	27	33.5 (33.3–34.5)	10	33.4 (33.0–33.9)	0.171
Cold pain (^o^C)	27	19.0 (12.7–22.5)	10	8.0 (0.8–16.0)	0.002
Heat pain (^o^C)	27	40.5 (39.4–43.7)	10	44.5 (39.6–45.9)	0.141
Method of levels					
Cold threshold (^o^C)	28	31.3 (30.5–31.8)	10	30.9 (27.4–31.8)	0.561
Heat threshold (^o^C)	28	32.4 (32.1–33.1)	10	32.8 (32.5–33.6)	0.474

^a^ Mann–Whitney *U* test.

#### Relationship of preoperative T-QST measurement with NRS and rescue medication

The preoperative cold {−0.426 [95% Confidence Interval (CI) −0.66 to −0.12]; *P* = 0.009} and heat [0.392 (95% CI 0.083–0.632); *P* = 0.016] pain thresholds (method of limits) were significantly related to the number of times an NRS ≥ 4 was reported during the first week postoperatively (Table 3), but not to the cumulative postoperative analgesic requirement (Table 4).

**Table 3:** Spearman's rho correlation between preoperative thermal-QST measurements and number of pain events (NRS ≥ 4) during one week postoperatively and thermal testing measurements.

Method of limits	*ρ*^a^ (95% CI)^b^ *N* = 37	*P*-value
Cold sensitivity	0.295 (−0.032 to 0.565)	0.076
Warmth sensitivity	−0.307 (−0.573 to −0.64)	0.064
Cold pain	−0.426 (−0.659 to −0.119)	0.009*
Heat pain	0.392 (0.078 to −0.635)	0.016**
Method of levels	*N* = 38	
Cold threshold	0.118 (−0.209 to 0.421)	0.481
Heat threshold	−0.002 (−0.321 to 0.317)	0.992

^a^ Spearman's rho.

^b^ CI = Confidence interval.

* Correlation is significant at the 0.01 level (2-tailed).

** Correlation is significant at the 0.05 level (2-tailed).

**Table 4:** Ordinal logistic regression between the number of rescue analgesics and a possible prediction of the thermal-QST measurements at start.

	Odds ratio	95% CI^b^	*p*-value
*N* rescue meds = 0^a^	1,870.8	2.130E-29 to 1.643E + 35	0.841
*N* rescue meds < 5^a^	18,443.3	2.080E-28 to 1.636E + 36	0.794
Cold sensitivity (^o^C)	0.871	0.373 to 2.030	0.748
Warmth sensitivity (^o^C)	0.528	0.140 to 1.987	0.345
Cold pain (^o^C)	0.900	0.798 to 1.015	0.086
Heat pain (^o^C)	0.792	0.622 to 1.008	0.058
Cold threshold (^o^C)	1.783	0.942 to 3.376	0.076
Heat threshold (^o^C)	2.369	1.034 to 5.425	0.041

^a^ Threshold parameters; 0 = no rescue medication; 1 = <5 times rescue medication and 2 = >5 times rescue medication was administered.

^b^ CI = Confidence interval.

#### Cognitive impairment and T-QST

Six out of the seven cognitively impaired participants (Vineland score of minus 2 SD) were able to finish all T-QST measurements, in one participant this was not possible.

Nevertheless, we noted that T-QST measurements and pain self-reporting are possible for most children with a minus 2 SD level on the Adaptive Behavior of the Vineland scale.

#### Scoliosis angle severity and correction

We found no evidence of a relationship between the degree of correction after scoliosis surgery and the risk for postoperative or chronic pain.

## Discussion

In this prospective, exploratory cohort study, preoperative, acute and chronic postoperative pain were evaluated in the same adolescents undergoing scoliosis surgery. We found that, 10 out of 39 (26%) adolescents patients had chronic postsurgical pain (CPSP) one year after surgery. More specifically, all patients experienced pain at moving and 2 also at rest. According to the DN4-7 items score, 4 patients experienced neuropathic pain (30). The 26% prevalence of chronic pain in this cohort was significantly lower than the 38% to 53% reported in previous studies, which in almost all cases concerned neuropathic pain (4, 39). Considering that almost 90% of our patients reported pain before surgery, and an important secondary outcome for surgery is chronic pain, the observed reduction in prevalence and severity of pain of one year after surgery is significant and suggests a positive effect on pain relief of scoliosis surgery. At the same time a chronic pain prevalence of 26% is unsatisfactory.

Our study also showed, that despite a structured postoperatively pain protocol, many patients still experienced significant pain in the direct postoperative period, for which additional clonidine and strong opioids were needed. This is disappointing, as we expected that a strict pain protocol with an epidural followed by a strong opioid would minimize acute pain. Interestingly, we did not find a relationship between patients in need for strong rescue medication and CPSP at one year postoperatively, which suggests that other biological or psychological factors play a role at different stages postoperatively.

To better understand, and potentially prevent or treat, chronic pain after scoliosis surgery, we explored the relationship between pain sensitivity, using T-QST and pain reporting pre- and postoperatively.

We found a correlation between preoperative T-QST values and moderate to severe acute pain after scoliosis surgery as well as with chronic pain. We found a relationship between the preoperative cold pain threshold and the persistence of chronic pain (*p* = 0.002) one year after scoliosis surgery. In contrast to our expectations this threshold was lower for patients with CPSP than for patients without CPSP, i.e., the CPSP-patients were less sensitive to cold pain. On the other hand and in line with our expectations, we found a positive correlation between the preoperative cold pain threshold (method of limits) and the number of times an NRS ≥ 4 was reported during the first week postoperatively.

Enhanced pain sensitivity caused by impaired pain modulation or delayed recovery from central sensitization might be a factor in the transition from acute to chronic pain (16, 40). However, pain thresholds measured postoperatively did not differ between our patients with and without CPSP. In contrast, Teles and colleagues showed with a more extended QST test, including a conditioning stimulus, impaired pain modulation by an inefficient pain inhibitory response in almost half of 94 patients with adolescent idiopathic scoliosis and chronic back pain (16). Unfortunately, they did not test the patients in relation to surgery. To limit the burden to the participants in our study, we did not include the use of a conditioning stimulus. In a review concerning different types of surgery in adults, thermal heat pain and temporal summation of pressure pain showed most association with acute or chronic pain after surgery (41). A more recent review and meta-analysis showed that more dynamic QST parameters i.e., temporal summation of pain and conditioned pain modulation have the most predictive value for chronification of pain (17). Both tests require extensive cooperation of patients specifically, if it has to be repeated multiple times in the postoperative course. Knowing our adolescent population well, we expected a high study dropout if we would include these extensive QST measurements and hence, pragmatically decided to more extensive testing. Therefore, in our study in adolescents we limited ourselves to T-QST testing. Still, considering our unexpected findings more research is recommended to test the hypothesis that patients with impaired pain modulation are at risk to develop chronic pain after surgery.

Furthermore, it would be worthwhile to test whether perioperative pain reducing interventions can influence the postoperative pain course. Several approaches have been suggested to prevent chronification of postoperative pain after spinal surgery, such as the use of epidural analgesia including opioids, non-steroidal anti-inflammatory drugs, gabapentinoids, and NMDA-antagonists (42). The use of intravenous ketamine, as an NMDA-antagonist, to reduce postoperative opioid consumption until six weeks postoperatively has shown benefits in lumbar back surgery in adults (43), whereas others did not find this benefit in scoliosis surgery in adolescents (44). Other previous studies have focused on the use of perioperative gabapentin and showed contradicting results on postoperative opioid consumption without an effect on chronic pain (4, 45, 46). Differences in pre-operative pain sensitivity in the patient populations studied may have contributed to these disparate results. Personalized pain treatment based on pre-operative pain sensitivity may be another option to improve pain outcomes after scoliosis surgery.

To further individualize pain treatment for children with scoliosis, a much larger prospective, multicenter multinational cohort may needed; its design could be guided based on the results from our prospective cohort results and those of retrospective studies. We believe our data support the inclusion of patients with non-idiopathic scoliosis in such studies, either as part of the larger cohort or as subgroups.

### Limitations

We did not perform a power analysis before start of the study, as this was an exploratory study aimed to collect data to guide the design of future prospective studies. As this was a very time-intensive prospective study with one year follow-up and an explorative aim, we used a convenience cohort.

The small number of participants is an important limitation of the study. Especially, the group of cognitively impaired patients was too small to make definite conclusions on the relationship between T-QST testing and postoperative pain for this specific patient population.

The T-QST measurements focused on heat and cold pain thresholds. It would have been of additional value if we had also determined the mechanical detection threshold using von Frey filaments, as described by Rolke et al. (19) or tests focussing on Temporal Summation or Conditioned Pain Modulation (17). Due to practical limitations of our clinical setting this was not an option.

Lastly, we used the DN4 test to identify neuropathic pain. This test has neither been validated for the pediatric population nor for patients with a mild cognitive impairment (VABS minus 2 SD), but is also used in other studies concerning scoliosis surgery in adolescents (4).

Still, the study participants were able to give a good description of the nature of their pain. Hence, we considered the use of this tool warranted for diagnosing neuropathic pain.

## Conclusions

Despite thorough perioperative pain medication, 26% of the participants in this exploratory study experienced CPSP one year after scoliosis surgery, some with neuropathic characteristics. We found some evidence for a correlation between preoperative heat/cold pain thresholds and acute postoperative pain, as well as chronic pain. The inverse correlation between the preoperative cold pain threshold and the development of chronic pain after scoliosis surgery remains unexplained. Nevertheless, we believe our exploratory data show potential for QST testing to improve our understanding and prevention of chronic pain in this population.

## Data Availability

The raw data supporting the conclusions of this article will be made available by the authors, without undue reservation.

## Supplementary material

Supplementary Table S1 Median pain assessment per visit, preoperative (pretest) and at postoperative controls.

Supplementary Table S2 T-QST values per visit, preoperative (baseline) and at postoperative controls.

## References

[1] Kehlet H Jensen TS Woolf CJ. Persistent postsurgical pain: risk factors and prevention. Lancet (London, England). (2006) 367(9522):1618–25. 10.1016/S0140-6736(06)68700-X 16698416

[2] Werner MU Kongsgaard UEI. Defining persistent post-surgical pain: is an update required? Br J Anaesth. (2014) 113(1):1–4. 10.1093/bja/aeu012 24554546

[3] Williams G Howard RF Liossi C. Persistent postsurgical pain in children and young people: prediction, prevention, and management. Pain Rep. (2017) 2(5):e616. 10.1097/PR9.0000000000000616 29392231 PMC5777679

[4] Julien-Marsollier F David R Hilly J Brasher C Michelet D Dahmani S. Predictors of chronic neuropathic pain after scoliosis surgery in children. Scand J Pain. (2017) 17:339–44. 10.1016/j.sjpain.2017.09.002 28958698

[5] Lavand'homme P. Transition from acute to chronic pain after surgery. Pain. (2017) 158(Suppl 1):S50–s4. 10.1097/j.pain.0000000000000809 28134653

[6] Nikolajsen L Brix LD. Chronic pain after surgery in children. Curr Opin Anaesthesiol. (2014) 27(5):507–12. 10.1097/ACO.0000000000000110 25051264

[7] Charalampidis A Rundberg L Möller H Gerdhem P. Predictors of persistent postoperative pain after surgery for idiopathic scoliosis. J Child Orthop. (2021) 15(5):458–63. 10.1302/1863-2548.15.210090 34858532 PMC8582608

[8] Page MG Campbell F Isaac L Stinson J Katz J. Parental risk factors for the development of pediatric acute and chronic postsurgical pain: a longitudinal study. J Pain Res. (2013) 6:727–41. 10.2147/JPR.S51055 24109194 PMC3792832

[9] Fortier MA Chou J Maurer EL Kain ZN. Acute to chronic postoperative pain in children: preliminary findings. J Pediatr Surg. (2011) 46(9):1700–5. 10.1016/j.jpedsurg.2011.03.074 21929977

[10] Sieberg CB Simons LE Edelstein MR DeAngelis MR Pielech M Sethna N Pain prevalence and trajectories following pediatric spinal fusion surgery. J Pain. (2013) 14(12):1694–702. 10.1016/j.jpain.2013.09.005 24290449 PMC3873090

[11] Wong GT Yuen VM Chow BF Irwin MG. Persistent pain in patients following scoliosis surgery. Eur Spine J. (2007) 16(10):1551–6. 10.1007/s00586-007-0361-7 17410382 PMC2078302

[12] Monitto CL Hsu A Gao S Vozzo PT Park PS Roter D Opioid prescribing for the treatment of acute pain in children on hospital discharge. Anesth Analg. (2017) 125(6):2113–22. 10.1213/ANE.0000000000002586 29189368 PMC5728167

[13] Li MM Ocay DD Teles AR Ingelmo PM Ouellet JA Page MG Acute postoperative opioid consumption trajectories and long-term outcomes in pediatric patients after spine surgery. J Pain Res. (2019) 12:1673–84. 10.2147/JPR.S191183 31190974 PMC6536124

[14] Landman Z Oswald T Sanders J Diab M. Prevalence and predictors of pain in surgical treatment of adolescent idiopathic scoliosis. Spine. (2011) 36(10):825–9. 10.1097/BRS.0b013e3181de8c2b 21192302

[15] Sanders JO Carreon LY Sucato DJ Sturm PF Diab M. Preoperative and perioperative factors effect on adolescent idiopathic scoliosis surgical outcomes. Spine. (2010) 35(20):1867–71. 10.1097/BRS.0b013e3181efa6f5 20802382

[16] Teles AR Ocay DD Bin Shebreen A Tice A Saran N Ouellet JA Evidence of impaired pain modulation in adolescents with idiopathic scoliosis and chronic back pain. Spine J. (2019) 19(4):677–86. 10.1016/j.spinee.2018.10.009 30343045

[17] Petersen KK Vaegter HB Stubhaug A Wolff A Scammell BE Arendt-Nielsen L The predictive value of quantitative sensory testing: a systematic review on chronic postoperative pain and the analgesic effect of pharmacological therapies in patients with chronic pain. Pain. (2021) 162(1):31–44. 10.1097/j.pain.0000000000002019 32701654

[18] Mainka T Maier C Enax-Krumova EK. Neuropathic pain assessment: update on laboratory diagnostic tools. Curr Opin Anaesthesiol. (2015) 28(5):537–45. 10.1097/ACO.0000000000000223 26263122

[19] Rolke R Magerl W Campbell KA Schalber C Caspari S Birklein F Quantitative sensory testing: a comprehensive protocol for clinical trials. Eur J Pain (London, England). (2006) 10(1):77–88. 10.1016/j.ejpain.2005.02.003 16291301

[20] Treede RD. The role of quantitative sensory testing in the prediction of chronic pain. Pain. (2019) 160(Suppl 1):S66–s9. 10.1097/j.pain.0000000000001544 31008852

[21] von Elm E Altman DG Egger M Pocock SJ Gøtzsche PC Vandenbroucke JP. The strengthening the reporting of observational studies in epidemiology (STROBE) statement: guidelines for reporting observational studies. J Clin Epidemiol. (2008) 61(4):344–9. 10.1016/j.jclinepi.2007.11.008 18313558

[22] Castarlenas E Jensen MP von Baeyer CL Miró J. Psychometric properties of the numerical rating scale to assess self-reported pain intensity in children and adolescents: a systematic review. Clin J Pain. (2017) 33(4):376–83. 10.1097/AJP.0000000000000406 27518484

[23] von Baeyer CL. Children’s self-report of pain intensity: what we know, where we are headed. Pain Res Manag. (2009) 14(1):39–45. 10.1155/2009/259759 19262915 PMC2706563

[24] von Baeyer CL. Numerical rating scale for self-report of pain intensity in children and adolescents: recent progress and further questions. Eur J Pain (London, England). (2009) 13(10):1005–7. 10.1016/j.ejpain.2009.08.006 19766028

[25] von Baeyer CL Uman LS Chambers CT Gouthro A. Can we screen young children for their ability to provide accurate self-reports of pain? Pain. (2011) 152(6):1327–33. 10.1016/j.pain.2011.02.013 21392889

[26] Hicks CL von Baeyer CL Spafford PA van Korlaar I Goodenough B. The faces pain scale-revised: toward a common metric in pediatric pain measurement. Pain. (2001) 93(2):173–83. 10.1016/S0304-3959(01)00314-1 11427329

[27] Bouhassira D Attal N Alchaar H Boureau F Brochet B Bruxelle J Comparison of pain syndromes associated with nervous or somatic lesions and development of a new neuropathic pain diagnostic questionnaire (DN4). Pain. (2005) 114(1–2):29–36. 10.1016/j.pain.2004.12.010 15733628

[28] Mathieson S Maher CG Terwee CB de Campos T F Lin CW. Neuropathic pain screening questionnaires have limited measurement properties. A systematic review. J Clin Epidemiol. (2015) 68(8):957–66. 10.1016/j.jclinepi.2015.03.010 25895961

[29] Van Seventer R Vos C Meerding W Mear I Le Gal M Bouhassira D Linguistic validation of the DN4 for use in international studies. Eur J Pain (London, England). (2010) 14(1):58–63. 10.1016/j.ejpain.2009.01.005 19282208

[30] van Seventer R Vos C Giezeman M Meerding WJ Arnould B Regnault A Validation of the Dutch version of the DN4 diagnostic questionnaire for neuropathic pain. Pain Pract. (2013) 13(5):390–8. 10.1111/papr.12006 23113981

[31] van den Bosch GE van Dijk M Tibboel D Valkenburg AJ. Thermal quantitative sensory testing in healthy Dutch children and adolescents standardized test paradigm and Dutch reference values. BMC Pediatr. (2017) 17(1):77. 10.1186/s12887-017-0827-7 28302148 PMC5356312

[32] de Graaf J Valkenburg AJ Tibboel D van Dijk M. Thermal detection thresholds in 5-year-old preterm born children; IQ does matter. Early Hum Dev. (2012) 88(7):487–91. 10.1016/j.earlhumdev.2011.12.006 22245233

[33] Defrin R Pick CG Peretz C Carmeli E. A quantitative somatosensory testing of pain threshold in individuals with mental retardation. Pain. (2004) 108(1–2):58–66. 10.1016/j.pain.2003.12.003 15109508

[34] Asher MA Min Lai S Burton DC. Further development and validation of the scoliosis research society (SRS) outcomes instrument. Spine. (2000) 25(18):2381–6. 10.1097/00007632-200009150-00018 10984792

[35] Haher TR Gorup JM Shin TM Homel P Merola AA Grogan DP Results of the scoliosis research society instrument for evaluation of surgical outcome in adolescent idiopathic scoliosis. A multicenter study of 244 patients. Spine. (1999) 24(14):1435–40. 10.1097/00007632-199907150-00008 10423788

[36] Society SR. Scoliosis Research Society. Adolescent idiopathic scoliosis. (2009). Available at: http://www.srs.org/professionals/education/adolescent/idiopathic/

[37] Sparrow S Cicchetti D Balla D. Vineland Adaptive Behavior Scales, Second Edition (Vineland-II); A Measure of Adaptive Behavior from Birth to Adulthood. Vineland-II, Vineland Adaptive Behavior Scales Expanded Interview Form Manual. Bloomington: NCS Pearson Inc (2005).

[38] Dijkxhoorn Y Verhaar L. Gebruikershandleiding Proefversie Vineland-II; Nederlandse Vertaling. Vineland Adaptive Behavior Scales, Second Edition (Vineland-II). Leiden: Leiden University Press (2008).

[39] Chidambaran V Ding L Moore DL Spruance K Cudilo EM Pilipenko V Predicting the pain continuum after adolescent idiopathic scoliosis surgery: a prospective cohort study. Eur J Pain (London, England). (2017) 21(7):1252–65. 10.1002/ejp.1025 PMC5541247 28346762

[40] Perry M Starkweather A Baumbauer K Young E. Factors leading to persistent postsurgical pain in adolescents undergoing spinal fusion: an integrative literature review. J Pediatr Nurs. (2018) 38:74–80. 10.1016/j.pedn.2017.10.013 29167085

[41] Sangesland A Støren C Vaegter HB. Are preoperative experimental pain assessments correlated with clinical pain outcomes after surgery? A systematic review. Scand J Pain. (2017) 15:44–52. 10.1016/j.sjpain.2016.12.002 28850344

[42] McGreevy K Bottros MM Raja SN. Preventing chronic pain following acute pain: risk factors, preventive strategies, and their efficacy. Eur J Pain Suppl. (2011) 5(2):365–72. 10.1016/j.eujps.2011.08.013 22102847 PMC3217302

[43] Loftus RW Yeager MP Clark JA Brown JR Abdu WA Sengupta DK Intraoperative ketamine reduces perioperative opiate consumption in opiate-dependent patients with chronic back pain undergoing back surgery. Anesthesiology. (2010) 113(3):639–46. 10.1097/ALN.0b013e3181e90914 20693876

[44] Pestieau SR Finkel JC Junqueira MM Cheng Y Lovejoy JF Wang J Prolonged perioperative infusion of low-dose ketamine does not alter opioid use after pediatric scoliosis surgery. Paediatr Anaesth. (2014) 24(6):582–90. 10.1111/pan.12417 24809838

[45] Mayell A Srinivasan I Campbell F Peliowski A. Analgesic effects of gabapentin after scoliosis surgery in children: a randomized controlled trial. Paediatr Anaesth. (2014) 24(12):1239–44. 10.1111/pan.12524 25230144

[46] Rusy LM Hainsworth KR Nelson TJ Czarnecki ML Tassone JC Thometz JG Gabapentin use in pediatric spinal fusion patients: a randomized, double-blind, controlled trial. Anesth Analg. (2010) 110(5):1393–8. 10.1213/ANE.0b013e3181d41dc2 20418301

