# Supplementary material for: Pain during the first year after scoliosis surgery in adolescents, an exploratory, prospective cohort study
Source: Front Pediatr. 2024 Jan 19;12:1293588. doi: 10.3389/fped.2024.1293588 (PMC10834739; doi:10.3389/fped.2024.1293588)
Supplement: Supplementary Table S1 [file Table1.docx]

| **Supplement 1:** Pain assessment per visit | | | | | |
| --- | --- | --- | --- | --- | --- |
|  | pretest  N=39  Median (IQR)^a^ | 6 weeks PO^b^  N =38  Median (IQR) | 3 months PO  N =39  Median (IQR) | 6 months PO  N =38  Median (IQR) | 12 months PO  N =38  Median (IQR) |
| NRS present | 0 (0 to 3.0) | 0.5 (0 to 2.3) | 0 (0 to 1.0) | 0 (0 to 1.0) | 0 (0 to 1.0) |
| NRS movement | 5.0 (3.8 to 7.3) | 3.0 (2.0 to 5.0) | 3.0 (1.0 to 5.0) | 2.0 (2.0 to 4.0) | 2.0 (0 to 4.0) |
| NRS rest | 0 | 0 (0 to 1.0) | 0 | 0 | 0 |
| NRS least | - | 0 (0 to 0.03) |  | 0 | 0 |
| NRS worst | - | 4.0 (2.0 to 5.0) |  | 2.0 (0.8 to 5.3) | 2.5 (1.0 to 2.3) |
| ^a^IQR = interquartile range; ^b^PO = postoperative | | | | | |
