# Supplementary material for: Pain during the first year after scoliosis surgery in adolescents, an exploratory, prospective cohort study
Source: Front Pediatr. 2024 Jan 19;12:1293588. doi: 10.3389/fped.2024.1293588 (PMC10834739; doi:10.3389/fped.2024.1293588)
Supplement: Supplementary Table S2 [file Table2.docx]

| **Supplement 2:** T-QST per visit | | | | | |
| --- | --- | --- | --- | --- | --- |
|  | Baseline  N = 38 | 6 weeks  N = 37 | 3 months  N = 37 | 6 months  N = 35 | 12 months  N = 38 |
| Detection threshold cold (MLI), mean °C (SD) | 29.9 (0.7) | 29.2 (0.8) | 28.9 (0.8) | 28.9 (0.7) | 28.5 (1.0) |
| Detection threshold warmth (MLI), °C (SD) | 33.8 (0.5) | 34.3 (0.5) | 34.5 (0.6) | 34.6 (0.6) | 34.6 (0.6) |
| Pain threshold cold (MLI), mean °C (SD) | 15.6 (2.6) | 17.6 (1.7) | 16.7 (1.5) | *(34)15.6 (1.8) | 16.1 (1.6) |
| Number of times 0° C was reached, n (%) | 11 (28.9) | 4 (10.8) | 11 (29.7) | 6 (5.6) | 7 (10.5) |
| Pain threshold heat (MLI), mean °C (SD) | 41.8 (1.3) | 41.2 (1.1) | 42.5 (1.2) | *(34)42.9 (1.2) | 42.7 (1.2) |
| Number of times 50°C was reached, n (%) | 7 (18.8) | 4 (10.8) | 9 (24.3) | 8 (11.1) | 7 (10.5) |
| Patients | N = 39 |  |  |  |  |
| Detection threshold cold (MLE), mean °C (SD) | 30.5 (1.9) | 30.3 (2.3) | 30.4 (1.9) | 30.8 (1.5) | 30.4 (2.1) |
| Detection threshold warmth (MLE), mean °C (SD) | 33.2 (1.9) | 33.3 (2.1) | 33.4 (1.8) | 33.3 (1.3) | 33.1 (1.3) |
| MLI, method of limits; MLE, method of levels; SD, standard deviation. * It was not possible to measure pain threshold in one participant. | | | | | |
